# Whole-Genome Sequence of *Salmonella enterica* Serovar Enteritidis Phage Type 4, Isolated from a Brazilian Poultry Farm

**DOI:** 10.1128/genomeA.00340-16

**Published:** 2016-05-12

**Authors:** Guilherme Paier Milanez, Leandro Costa Nascimento, Adriane Holtz Tirabassi, Marcelo Zuanaze, Dália Prazeres Rodrigues, Gonçalo Amarante Guimarães Pereira, Marcelo Brocchi

**Affiliations:** a Department of Genetics, Evolution and Bioagents, Institute of Biology, University of Campinas, São Paulo, Brazil; b Genomics and Expression Laboratory (LGE), Department of Genetics, Evolution and Bioagents, Institute of Biology, University of Campinas, São Paulo, Brazil; c Central Laboratory of High Performance Technologies (LaCTAD), University of Campinas, São Paulo, Brazil; d Biovet Laboratory, Vargem Grande Paulista, São Paulo, Brazil; e Department of Bacteriology, Oswaldo Cruz Institute, FIOCRUZ, Rio de Janeiro, Brazil

## Abstract

The draft genome of *Salmonella enterica* serovar Enteritidis phage type 4 (PT4) strain IOC4647/2004, isolated from a poultry farm in São Paulo state, was obtained with high-throughput Illumina sequencing platform, generating 4,173,826 paired-end reads with 251 bp. The assembly of 4,804,382 bp in 27 scaffolds shows strong similarity to other *S*. Enteritidis strains.

## GENOME ANNOUNCEMENT

Nontyphoidal *Salmonella* (NTS) is one of the commonest bacterial pathogens causing gastrointestinal infection worldwide. *Salmonella enterica* serovar Enteritidis is the most prevalent serovar (1); it accounts for huge global disease burden and causes human gastroenteritis and bacteremia (2, 3). Since 1993, *S*. Enteritidis has been the main serovar underlying human infections isolated from poultry materials in Brazil. This serovar is still the cause of many losses faced by the national poultry industry (4).

Although our understanding of the host immune response to *S. enterica* infections has improved considerably, some important questions remain unanswered (5). Comparative analysis of different *Salmonella* genomes revealed that core regions resembled each other closely, and that continuous genetic re-assortment increased virulence and generated multiple-drug resistant isolates, which is of significant public health concern (6). An important feature among different *S*. Enteritidis isolates is the genetic similarity they share, which makes their discrimination by traditional genotyping methods difficult (7). Hence, sequencing samples from different locations worldwide is crucial to detect specific differences that might be related to factors driving the evolution of this pathogen.

Here we announce a whole-genome sequence of Brazilian *S*. Enteritidis phage type 4 strain IOC4746, considered a representative strain isolated in the state of São Paulo in 2004 (FIOCRUZ, unpublished data). DNA was isolated from overnight Luria-Broth culture using a DNA purification kit (Promega Corporation, Madison, WI, USA). Libraries were prepared with the Nextera mate-pair sample preparation kit and the TruSeq DNA PCR-free LT sample prep kit according to the manufacturer’s instructions (Illumina, San Diego, CA, USA). The genome was sequenced with the Illumina HiSeq2500 platform, which produced 4,173,826 paired-end reads with 251 bp. The reads were *de novo* assembled using SPAdes version 3.6.0 (8). Contigs larger than 1,000 bp were scaffolded using SSPACE version 2.0 (9), which gave 27 scaffolds and totaled 4,804,382 bp (largest: 1,549,687 bp; *N*_50_: 490,431 bp; mean: 177,940 bp).

The contigs were aligned with the reference *S*. Enteritidis strain P125109 using MAUVE (10), and an automated annotation was made with RAST (11). Two scaffolds containing 51,142 and 7,763 bp, respectively, perfectly aligned with the virulence plasmid of P125109, but it was not possible to close it. The alignment showed that this *S*. Enteritidis isolate presents high identity with the P125109 strain. A kmerfinder (12) confirmed this observation, and multilocus sequencing typing (13) indicated that strain IOC4746 is sequence type 11 (ST 11), the most common ST among “classic” *S*. Enteritidis isolates (14).

### Nucleotide sequence accession numbers.

The whole-genome sequence generated in this project has been deposited at DDBJ/ENA/GenBank under the accession no. LTDW00000000. The version described in this paper is the first version, LTDW01000000.
